# Real-World Germline Testing Patterns and Clinical Implications of HRR-Associated Germline Variants in Pancreatic Ductal Adenocarcinoma

**DOI:** 10.3390/cancers18162580

**Published:** 2026-08-11

**Authors:** Carmen Blanco Abad, Diego Casas Deza, Fátima Mocha Campillo, Natalia Pascual de la Fuente, Sofía Elena Ruffini Egea, Luis Gallart Caballero, Cristina Serrano Delgado, Carlos Alfonso Rivas Molina, María Dolores Miramar Gallart, María José Agustín Ferrández, Paula Gomila Pons, Sara Elena Campos Ramírez, Marta Gascón Ruiz, Eduardo Polo Marques, Roberto Antonio Pazo Cid

**Affiliations:** 1 Department of Medical Oncology, Miguel Servet University Hospital, 50012 Zaragoza, Spain; 2 ADIPOFAT Lab, Department of Gastroenterology, Miguel Servet University Hospital, Aragón Health Research Institute, 50012 Zaragoza, Spain; diegocasas8@gmail.com; 3 Department of Medicine, Psychiatry and Dermatology, Faculty of Medicine, University of Zaragoza, 50009 Zaragoza, Spain; 4 Clinical Genetics Unit, Miguel Servet University Hospital, 50012 Zaragoza, Spain; 5 Department of Hospital Pharmacy, Miguel Servet University Hospital, 50012 Zaragoza, Spain; 6 Department of Medical Oncology, Lozano Blesa University Clinical Hospital, 50009 Zaragoza, Spain

**Keywords:** pancreatic ductal adenocarcinoma, germline testing, homologous recombination repair, platinum-based chemotherapy, genetic counseling

## Abstract

**Background and Aim:** Germline testing (GT) is increasingly relevant in pancreatic ductal adenocarcinoma (PDAC) because of therapeutic and familial implications. We evaluated real-world GT uptake, the diagnostic yield of pathogenic/likely pathogenic germline variants (P/LP), and their clinical implications. **Methods:** We retrospectively evaluated 674 consecutive patients with PDAC. GT was performed using targeted single-gene testing or multigene panels. Survival analyses among patients receiving platinum-based chemotherapy according to P/LP germline variant status in HRR-associated genes were exploratory. **Results:** Overall, 28.9% of patients underwent GT, increasing to 45.9% in 2021–2025 (*p* < 0.001). Results were available for 194 patients; 28 (14.4%) harbored P/LP variants, most commonly *BRCA2* (4.6%) and *ATM* (3.1%). P/LP variants were identified in 6.8% of patients who did not meet classical referral criteria. Among the 28 carriers, 12 (42.9%) harbored *BRCA1/2* or *PALB2*, variants with established therapeutic relevance, while eight (28.6%) carried *ATM*, *FANCA*, or *FANCM,* variants with potential therapeutic relevance. Of these 20 patients, 11 (55.0%) received matched therapy, and treatment was modified because of the germline result in six (30.0%). In exploratory analyses, among platinum-treated patients, carriers of P/LP germline variants in HRR-associated genes had longer PFS than non-carriers (23.9 vs. 4.8 months; log-rank *p* = 0.017), although only six HRR-associated variant carriers were included in this platinum-treated survival analysis. **Conclusions:** GT remained underused despite increasing implementation. Early systematic GT may identify clinically relevant variants missed by selective referral strategies and inform treatment, genetic counseling, and cascade testing. Survival findings require prospective validation.

## Simple Summary

Pancreatic ductal adenocarcinoma is an aggressive cancer with limited treatment opportunities, making the identification of inherited genetic alterations clinically relevant. In this real-world study, we evaluated the implementation and clinical impact of germline testing in patients with pancreatic cancer. Testing increased over time but remained underused. Pathogenic or likely pathogenic germline variants were identified in a relevant proportion of tested patients, including some who did not meet traditional referral criteria based on personal or family history. Most variant carriers had alterations with established or potential therapeutic implications, although only a subset received matched treatment and fewer had their treatment specifically modified because of the germline result. Exploratory survival analyses suggested favorable outcomes among patients with homologous recombination repair-associated variants treated with platinum-based chemotherapy, but the small number of patients and the use of PARP inhibitors in some cases prevent firm conclusions about a platinum-specific effect. Overall, our findings support early and systematic germline testing to inform treatment decisions, genetic counseling, and cascade testing of relatives.

## 1. Introduction

Pancreatic ductal adenocarcinoma (PDAC) has a poor prognosis, with a 5-year overall survival (OS) rate of approximately 12% [1]. This is largely due to the high proportion of patients who present with advanced, unresectable disease [2]. In the metastatic setting, therapeutic options are limited, and only a minority of patients are able to receive subsequent lines of therapy beyond first-line treatment [3]. In this context, platinum-based chemotherapy regimens represent an important treatment option for selected patients, although their use is not universal [4,5,6].

A proportion of PDAC cases, estimated at 10–15%, are associated with an underlying hereditary predisposition, either due to the presence of germline pathogenic variants (GPVs) in cancer susceptibility genes or to familial aggregation consistent with familial pancreatic cancer [7,8]. Germline alterations in genes involved in DNA damage repair pathways, particularly homologous recombination repair (HRR), are recurrently identified in PDAC [9]. The reported prevalence of GPVs varies widely across studies, ranging from 4% to 21% [5,8,10,11,12,13,14,15], likely reflecting differences in patient selection, gene panels, and geographic distribution. In unselected populations, the prevalence appears to be lower, generally ranging from 6% to 11.5% [7,16,17,18].

The growing recognition of the clinical relevance of germline alterations has led to changes in germline testing (GT) recommendations. Current guidelines from the NCCN advocate universal GT for all patients diagnosed with PDAC, regardless of clinical or familial characteristics [19]. In contrast, European guidelines, including those from the European Society for Medical Oncology (ESMO), adopt a more selective approach, recommending *BRCA* testing for patients with metastatic PDAC to guide the selection of platinum-based chemotherapy and potential maintenance treatment with olaparib. Patients with a relevant family history or other high-risk features should undergo genetic counseling and may be considered for broader germline evaluation [20]. Despite these recommendations, the implementation of GT in routine clinical practice remains suboptimal [21,22,23,24].

The identification of GPVs has important implications not only for patients but also for their families. From a therapeutic perspective, tumors harboring alterations in HRR genes may be particularly sensitive to platinum-based chemotherapy and poly(ADP-ribose) polymerase (PARP) inhibitors, thereby offering opportunities for personalized treatment [2,4,25,26,27,28]. Additionally, cascade testing enables the identification of at-risk relatives who may benefit from surveillance programs aimed at early cancer detection [29]. Beyond established cancer-predisposition genes, emerging evidence also suggests that germline variants in genes associated with hereditary or chronic pancreatitis, particularly *PRSS1* and *CFTR*, may contribute to PDAC susceptibility [30].

In this multicenter study, we aimed to evaluate real-world GT uptake and determine the diagnostic yield of P/LP germline variants among tested patients with PDAC. We also assessed clinical and pathological factors associated with GT uptake and P/LP variant detection, as well as the frequency of P/LP variants among patients who did not meet classical referral criteria. Finally, we explored the therapeutic implications of identified germline variants and their association with survival outcomes.

## 2. Material and Methods

### 2.1. Inclusion Criteria and Study Design

This retrospective, multicenter study included 674 consecutive patients diagnosed between 2015 and 2025 with PDAC at Miguel Servet University Hospital (HUMS), San Jorge Hospital, Huesca, Barbastro Hospital, Alcañiz Hospital, and Obispo Polanco General Hospital. The study evaluated real-world uptake of GT, the diagnostic yield of P/LP germline variants, and their clinical and therapeutic implications.

Detailed clinical, pathological, molecular, treatment, and outcome data were retrospectively collected. Referral criteria were defined as follows: age ≤ 60 years at diagnosis; personal history of breast cancer or melanoma; and family history criteria including (i) three cases of pancreatic cancer within the same familial lineage; (ii) two first-degree relatives with pancreatic cancer; (iii) one case of pancreatic cancer plus one case of melanoma diagnosed before the age of 60; or (iv) three or more relatives affected by pancreatic, breast, ovarian, and/or prostate cancer.

During the early study period, GT was generally initiated after referral to the Clinical Genetics service. Following revision of the referral pathways in 2023, GT could be requested directly by the treating oncology team. Patients with P/LP germline findings were subsequently referred to Clinical Genetics for post-test counseling, hereditary cancer risk assessment, and cascade testing of relatives. This organizational change occurred alongside evolving European recommendations and increasing availability of germline testing.

Platinum-based chemotherapy was administered according to the standard institutional protocol applicable at the time of treatment. Platinum-containing regimens included FOLFIRINOX or modified FOLFIRINOX, NALIRIFOX, cisplatin plus gemcitabine, the SEQUENCE regimen, and FOLFOX6. Detailed drug doses and administration schedules are provided in Supplementary Table S1. Platinum exposure was defined as documented receipt of at least one administration of an oxaliplatin- or cisplatin-containing regimen. Dose modifications, treatment delays, and discontinuations were managed according to the applicable protocol and the patient’s clinical status. The actual number of cycles, cumulative dose intensity, individual dose reductions, treatment delays, and reasons for discontinuation were not systematically recorded.

Exposure to poly(ADP-ribose) polymerase inhibitors (PARPi) and the clinical setting in which they were administered were also recorded. During the study period, olaparib was not reimbursed by the Spanish National Health System for maintenance treatment of metastatic PDAC harboring germline *BRCA1/2* pathogenic variants. Therefore, although PARP inhibition was considered in potentially eligible patients, access in this setting was restricted to clinical trials or exceptional/compassionate-use pathways.

The primary objectives were to evaluate real-world GT uptake and determine the diagnostic yield of P/LP germline variants among tested patients with available results. Secondary objectives included: (i) to evaluate clinical factors associated with GT uptake and P/LP variant detection; (ii) to determine the frequency of P/LP germline variants among patients who did not meet classical referral criteria; (iii) to assess the therapeutic implications of identified germline variants, including receipt of matched therapy and treatment modification attributable to the germline result; and (iv) to explore survival outcomes according to P/LP germline variant status in HRR-associated genes among patients receiving platinum-based chemotherapy.

For the descriptive assessment of therapeutic impact, matched therapy was defined as receipt, at any time during the disease course, of a treatment considered biologically concordant with the identified germline alteration, including platinum-based chemotherapy, PARP inhibition, or genotype-directed clinical trial treatment. Receipt of matched therapy and modification of treatment specifically attributable to knowledge of the germline result were assessed as separate variables, since administration of a biologically matched treatment did not necessarily imply that treatment selection had been driven by the germline finding.

All procedures were performed in accordance with the ethical standards of the institutional and national research committees and complied with the principles outlined in the Declaration of Helsinki and its subsequent amendments. The study protocol was approved by the institutional ethics committee of HUMS (CEICA; C.I. PI25/340; approval date: 10 September 2025). Written informed consent was obtained from living patients included in the study.

### 2.2. Germline Testing and Molecular Analyses

GT was performed on peripheral blood samples at the Clinical Genetics Section of HUMS and at accredited external laboratories. Testing strategies ranged from targeted familial single-gene testing to multigene panels comprising up to 115 genes. Among patients evaluated using multigene panels, the median panel size was 12 genes. The most frequently used panel comprised 12 genes: *APC*, *ATM*, *BRCA1*, *BRCA2*, *CDKN2A*, *MLH1*, *MRE11A*, *MSH2*, *PALB2*, *PMS2*, *STK11*, and *TP53*. In one patient, targeted single-gene testing for *BRCA2* was performed because a pathogenic germline *BRCA2* variant had previously been identified in a relative; testing confirmed the presence of the same familial variant in the patient. The exact gene composition and the main technical characteristics of the different testing strategies are detailed in Supplementary Tables S2 and S3, respectively.

For multigene panel testing, next-generation sequencing (NGS) was performed on DNA extracted from peripheral blood lymphocytes. Library preparation and target enrichment were carried out using the NGS Clinical STARlet system (Hamilton Bonaduz AG, Bonaduz, Switzerland), Illumina DNA Prep with Enrichment Dx (Illumina, Inc., San Diego, CA, USA), and the Illumina Exome 2.5 plus Twist Bioscience for Illumina Mitochondrial Panel (Twist Bioscience Corporation, South San Francisco, CA, USA), or other commercially available validated workflows, according to the laboratory and testing period. Sequencing was performed on the Illumina NovaSeq 6000 Dx platform (Illumina, Inc., San Diego, CA, USA) or on other validated NGS platforms according to each laboratory’s protocol. Bioinformatic processing, variant calling, and interpretation were conducted using VarSome Clinical (CE-IVD, Saphetor SA, Lausanne, Switzerland) and/or platform-specific analysis pipelines.

Sequencing depth was considered adequate to ensure reliable variant detection across the targeted regions, including exon–intron boundaries. Platform-specific coverage thresholds and analytical characteristics are detailed in Supplementary Tables S2 and S3. Identified variants were interpreted and classified according to established international recommendations, including those of the American College of Medical Genetics and Genomics and the Association for Molecular Pathology (ACMG/AMP). Only variants classified as pathogenic or likely pathogenic (P/LP) were considered for the purposes of this study. Clinically relevant variants were confirmed by Sanger sequencing when indicated according to the corresponding laboratory protocol, while clinically relevant copy number variants (CNVs) were confirmed by multiplex ligation-dependent probe amplification and/or comparative genomic hybridization array when indicated and according to the applicable laboratory workflow.

For the exploratory survival analyses, HRR-associated genes were defined as *BRCA1*, *BRCA2*, *PALB2*, *ATM*, *FANCA*, and *FANCM*. P/LP variants in *BRCA1*, *BRCA2*, and *PALB2* were considered to have established therapeutic relevance, whereas variants in *ATM*, *FANCA*, and *FANCM* were classified as having potential therapeutic relevance, based on their involvement in HRR or related DNA damage response (DDR) pathways and the more limited clinical evidence supporting treatment selection. This classification did not constitute a tumor-level diagnosis of homologous recombination deficiency (HRD).

No systematic somatic second-hit analysis, loss-of-heterozygosity assessment, biallelic inactivation analysis, genomic scar score, mutational signature analysis, or functional HRD assay was performed. However, in the patient harboring a pathogenic germline FANCM variant, tumor comprehensive genomic profiling by NGS (Foundation Medicine, Inc., Cambridge, MA, USA) was also performed and reported an HRD-positive status.

### 2.3. Statistical Analysis

Categorical variables were expressed as absolute frequencies and percentages. Quantitative variables were expressed as mean ± standard deviation (SD) or median and interquartile range (IQR), depending on the distribution of the data. Normality was assessed using the Shapiro–Wilk test. For comparisons between groups, Student’s *t*-test, Welch’s *t*-test, or the Mann–Whitney U test was used for continuous variables, as appropriate, and the Chi-square test or Fisher’s exact test for categorical variables. Univariable and multivariable binary logistic regression analyses were used to evaluate factors associated with GT uptake and with the detection of P/LP germline variants, with results reported as odds ratios (ORs) and *95% CIs*. All tests were two-sided, and 95% confidence intervals (CIs) were calculated for the main estimates. Statistical significance was set at *p* < 0.05.

Survival analyses were exploratory and were restricted to patients with metastatic disease for treatment-related comparisons. Survival curves were estimated using the Kaplan–Meier method and compared using the log-rank test. Among platinum-treated patients, progression-free survival (PFS) was calculated from initiation of the first platinum-containing regimen administered in the metastatic setting to disease progression or death from any cause, whichever occurred first. Patients without disease progression or death at the time of last follow-up were censored.

Overall survival (OS) was defined as the time from initiation of first-line systemic treatment for advanced disease to death from any cause or last follow-up. For survival analyses among patients receiving platinum-based chemotherapy, patients were classified according to the presence or absence of P/LP germline variants in HRR-associated genes. The specific platinum-containing regimen and treatment line were recorded.

Cox proportional hazards regression was used to estimate associations with PFS and OS, with results reported as hazard ratios (HRs) and corresponding *95% CIs*. For PFS among patients receiving platinum-based chemotherapy in the metastatic setting, univariable analyses were performed and a multivariable model including HRR-associated germline variant status, age, and treatment line was fitted. Given the small number of HRR-associated variant carriers, OS Cox analyses were restricted to univariable models.

All analyses were performed using jamovi (version 2.7.12.0; jamovi project, Sydney, Australia, https://www.jamovi.org (accessed on 3 March 2026).

## 3. Results

### 3.1. Referral Patterns

**Figure 1:**
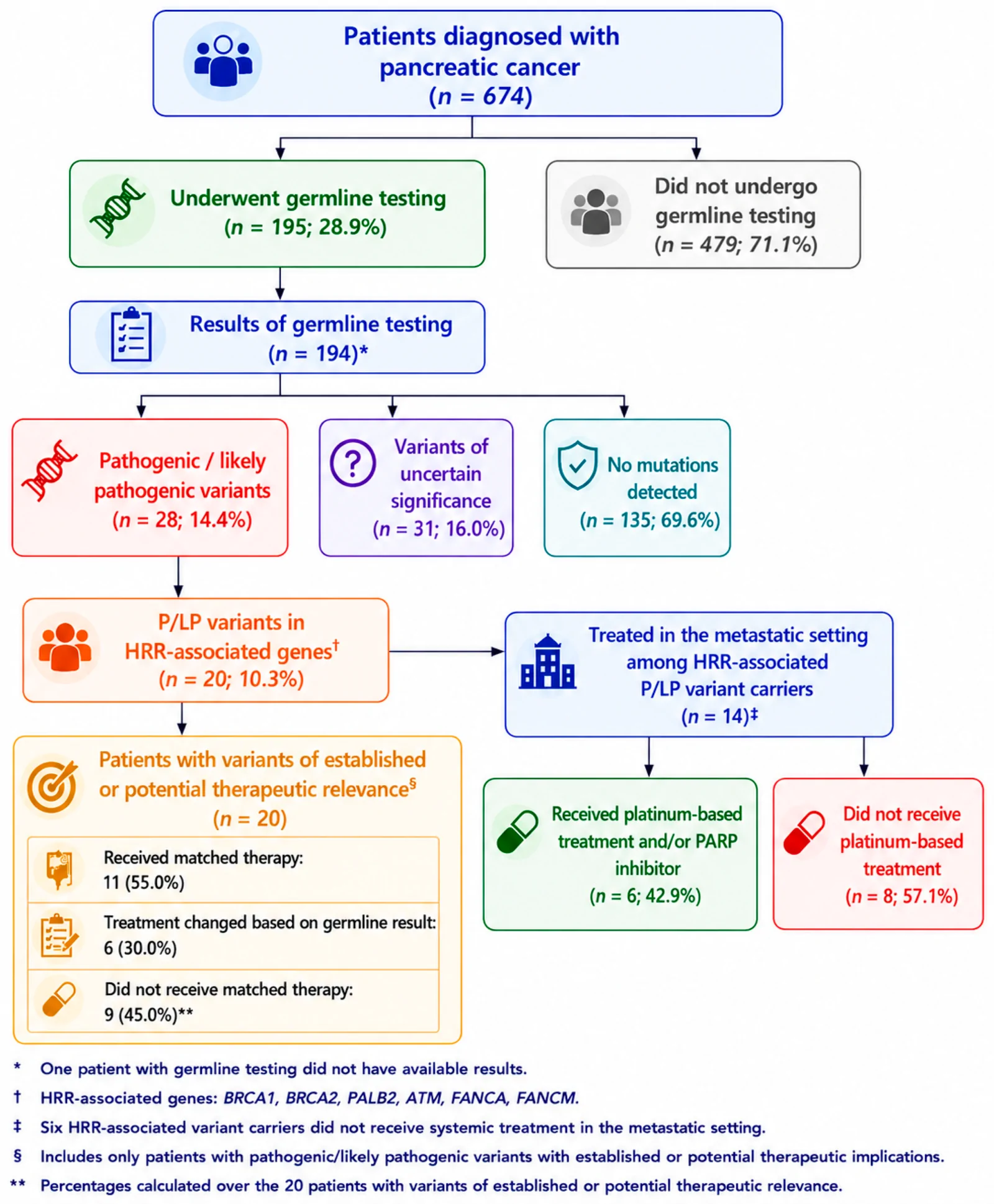
Flow diagram of patient inclusion, germline testing results, treatment exposure, and therapeutic implications.

Overall, 674 consecutive patients with PDAC were included. Of these, 349 (51.8%) were male and 325 (48.2%) were female. The mean age at diagnosis was 66.7 years, with a range of 35–89 years. The study flowchart, including patient selection, GT results, and exposure to platinum-based chemotherapy, is shown in Figure 1. Overall, 195/674 patients (28.9%; *95%* CI, 25.5–32.4%) underwent GT. Among tested patients, 88 (45.1%) did not fulfill any of the classical referral criteria. Regarding specific indications, 69 patients (35.4%) met the age criterion (≤60 years), 36 (18.5%) had a relevant family history, and 17 (8.7%) had a personal history of cancer associated with an increased risk of PDAC. These referral criteria were not mutually exclusive.

The proportion of patients who did not meet any predefined classical referral criteria was significantly higher among untested than tested patients (74.7% vs. 45.1%, *p* < 0.001). Among patients whose only indication for GT was the universal-testing recommendation (i.e., those who did not meet any classical referral criterion), only 19.8% underwent GT, whereas the majority (80.2%) remained untested. Conversely, 121 patients (25.3%) who did not undergo testing fulfilled at least one classical referral criterion. Baseline characteristics according to GT status are summarized in Supplementary Table S4.

In univariable analyses, family-history and personal-history referral criteria were significantly more frequent among tested than untested patients (18.5% vs. 0.6%, *p* < 0.001, and 8.7% vs. 1.0%, *p* < 0.001, respectively). Similarly, patients aged ≤60 years represented a higher proportion of the tested than the untested group (35.4% vs. 22.8%, *p* < 0.001).

In multivariable analysis, age ≤ 60 years (OR 2.10, *95%* CI, 1.40–3.10), family history referral criteria (OR 40.30, *95%* CI, 12.10–134.20), and personal history of cancer (OR 11.80, *95%* CI, 4.10–33.70) were independently associated with a higher likelihood of undergoing GT. In contrast, in a separate model, having no classical referral criterion and therefore being eligible solely on the basis of universal-testing recommendations was associated with a lower probability of undergoing GT (OR 0.28, *95%* CI, 0.20–0.40).

**Figure 2:**
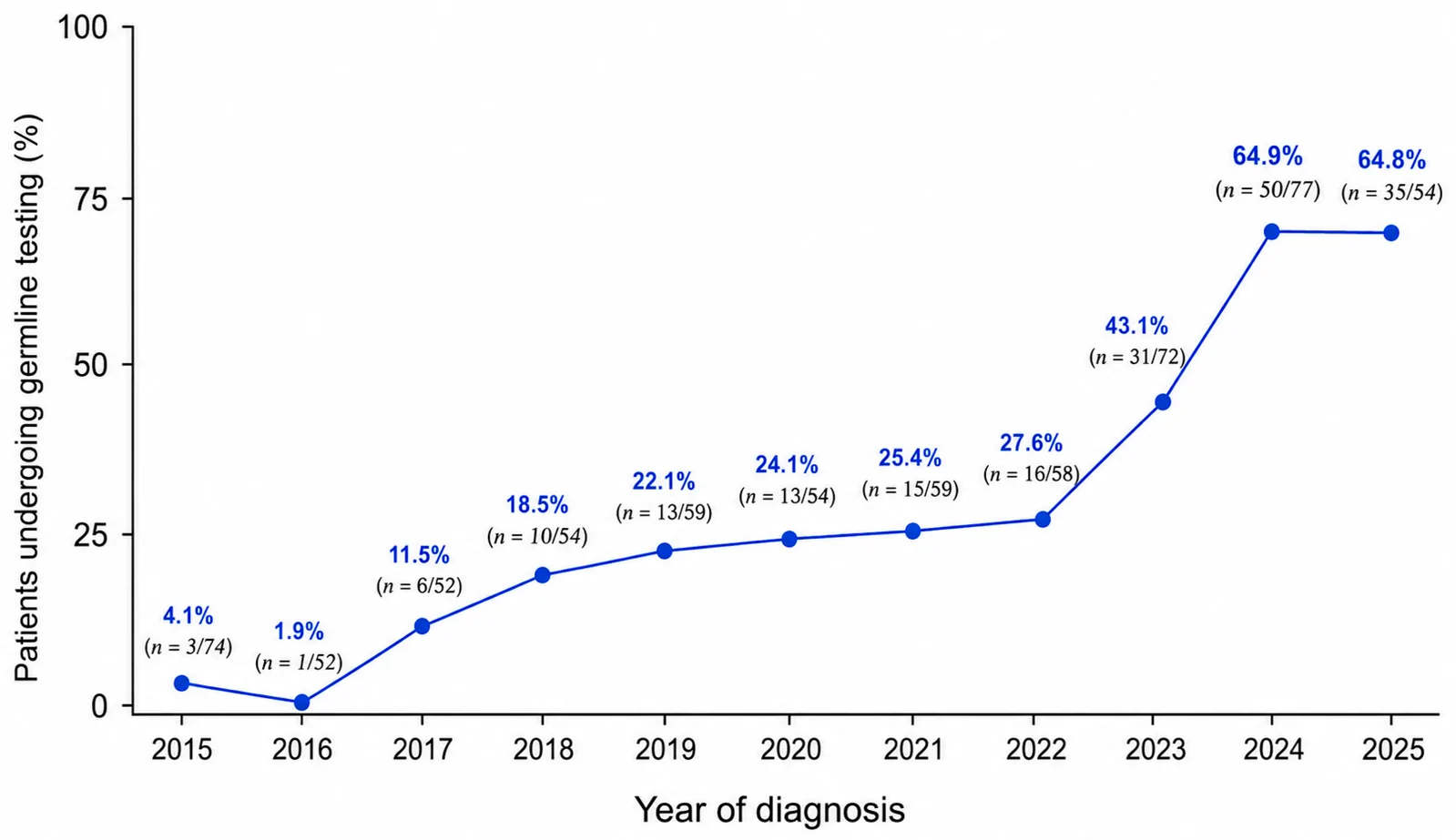
Annual proportion of patients with pancreatic ductal adenocarcinoma undergoing germline testing from 2015 to 2025.

The proportion of patients undergoing GT increased significantly over time, from 5.6% in 2015–2017 to 21.6% in 2018–2020 and 45.9% in 2021–2025 (*p* < 0.001), as shown in Figure 2. This increase coincided with updated guideline recommendations, revisions to referral pathways, greater testing availability, and the transition from a genetics-led referral pathway to direct GT initiation by the treating oncology team.

### 3.2. Diagnostic Yield of P/LP Germline Variants and Association Between Referral Criteria and Variant Detection

Among the 194 tested patients with an available germline result, 28 harbored a P/LP germline variant, corresponding to a diagnostic yield of 14.4% (*95%* CI, 10.0–20.1%), while 31 individuals (16.0%) harbored variants of uncertain significance (VUS).

**Figure 3:**
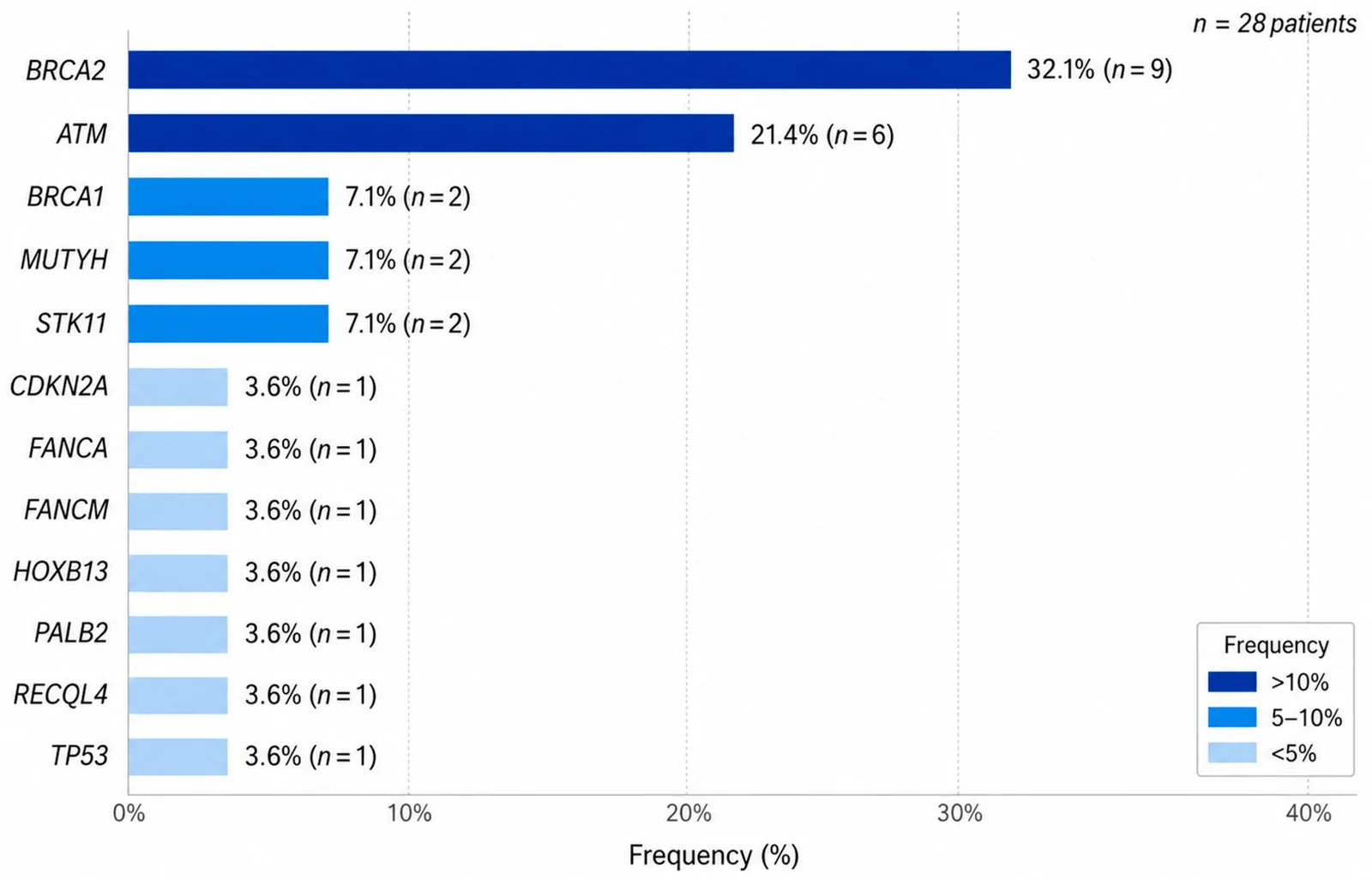
Distribution of pathogenic or likely pathogenic germline variants identified in patients with pancreatic ductal adenocarcinoma. Percentages were calculated among the 28 patients harboring P/LP germline variants.

Among patients with available germline results, the most frequently altered genes were *BRCA2* (*n* = 9, 4.6%) and *ATM* (*n* = 6, 3.1%). Other recurrent alterations included *BRCA1*, *STK11*, and *MUTYH* (each *n* = 2, 1.0%). Variants in additional genes, such as *PALB2*, *CDKN2A*, *TP53*, and several others, were identified in single cases. No variant reclassification was observed. The distribution of P/LP germline variants identified is shown in Figure 3.

The presence of a family-history referral criterion or personal-history referral criterion was significantly associated with a higher likelihood of identifying P/LP variants (30.6% vs. 10.8%, *p* = 0.002; OR 3.65, *95%* CI, 1.53–8.71; and 41.2% vs. 11.9%, *p* = 0.001; OR 5.20, *95%* CI, 1.79–15.1, respectively). In contrast, age at diagnosis ≤60 years was not associated with an increased P/LP variant detection rate (14.5% vs. 14.4%, *p* = 0.990; OR 1.01, *95%* CI, 0.44–2.32).

In multivariable analysis, family-history and personal-history referral criteria were independently associated with a higher likelihood of harboring P/LP germline variants (OR 5.00, *95%* CI, 1.91–13.0, *p* = 0.001; and OR 6.85, *95%* CI, 2.07–22.7, *p* = 0.002, respectively). In contrast, age at diagnosis, sex, and metastatic status were not significantly associated with P/LP variant detection.

Notably, 6.8% of patients who did not meet classical referral criteria (6/88) were found to carry P/LP germline variants, underscoring the potential limitations of selective testing strategies. These variants involved *ATM* in three patients and *BRCA2*, *CDKN2A*, and *TP53* in one patient each. Furthermore, 49 of 195 patients who underwent GT (25.1%) died before their germline results became available.

### 3.3. Therapeutic Implications and Survival Outcomes According to P/LP Germline Variant Status in HRR-Associated Genes

Among the 28 patients harboring P/LP germline variants, 12 patients (42.9% of the overall P/LP cohort) harbored variants in *BRCA1*, *BRCA2*, or *PALB2*, for which stronger clinical evidence supports treatment selection in PDAC [25,27,31,32]. An additional eight patients (28.6%) harbored P/LP variants in *ATM*, *FANCA*, or *FANCM*, which were considered to have potential therapeutic relevance based on their involvement in HRR/DDR pathways, although the supporting clinical evidence is more limited [31]. Overall, 20/28 patients (71.4%) therefore harbored germline alterations with either established or potential therapeutic implications. The therapeutic impact of GT was assessed separately according to receipt of matched therapy and modification of treatment specifically attributable to knowledge of the germline result. Patient-level details regarding therapeutic relevance, matched therapy, treatment modification, platinum exposure, PARPi use, and reasons for not receiving matched therapy are provided in Supplementary Table S5.

Among patients with variants of established or potential therapeutic relevance, 11/20 (55.0%) received matched therapy, corresponding to 39.3% of all P/LP carriers. All 11 received platinum-based chemotherapy, and two patients with germline *BRCA1/2* variants subsequently received a PARP inhibitor. Treatment was specifically modified based on the germline result in 6/20 patients (30.0%; 21.4% of all P/LP carriers). Nine patients did not receive matched therapy, mainly because of clinical frailty or platinum contraindication (*n* = 4), availability of the germline result after death (n = 3), ongoing benefit from another treatment (*n* = 1), or clinical deterioration following progression on ongoing treatment, precluding subsequent platinum therapy (*n* = 1).

Among the 195 patients who underwent GT, 188 received systemic treatment for metastatic disease. Of these, 53 patients (28.2%) received a platinum-based regimen, whereas 135 (71.8%) did not. The remaining seven patients had not received systemic treatment for metastatic disease.

Among the 53 patients who received platinum-based chemotherapy, 23 (43.4%) received FOLFIRINOX or mFOLFIRINOX, 15 (28.3%) received NALIRIFOX, 7 (13.2%) received FOLFOX6, 4 (7.5%) received cisplatin plus gemcitabine, and 4 (7.5%) received the SEQUENCE regimen. Platinum-containing therapy was administered in the first line in 33 patients (62.3%), in the second line in 11 (20.8%), and in the third line in 9 (17.0%). The distribution of individual regimens according to treatment line is provided in Supplementary Table S6.

**Figure 4:**
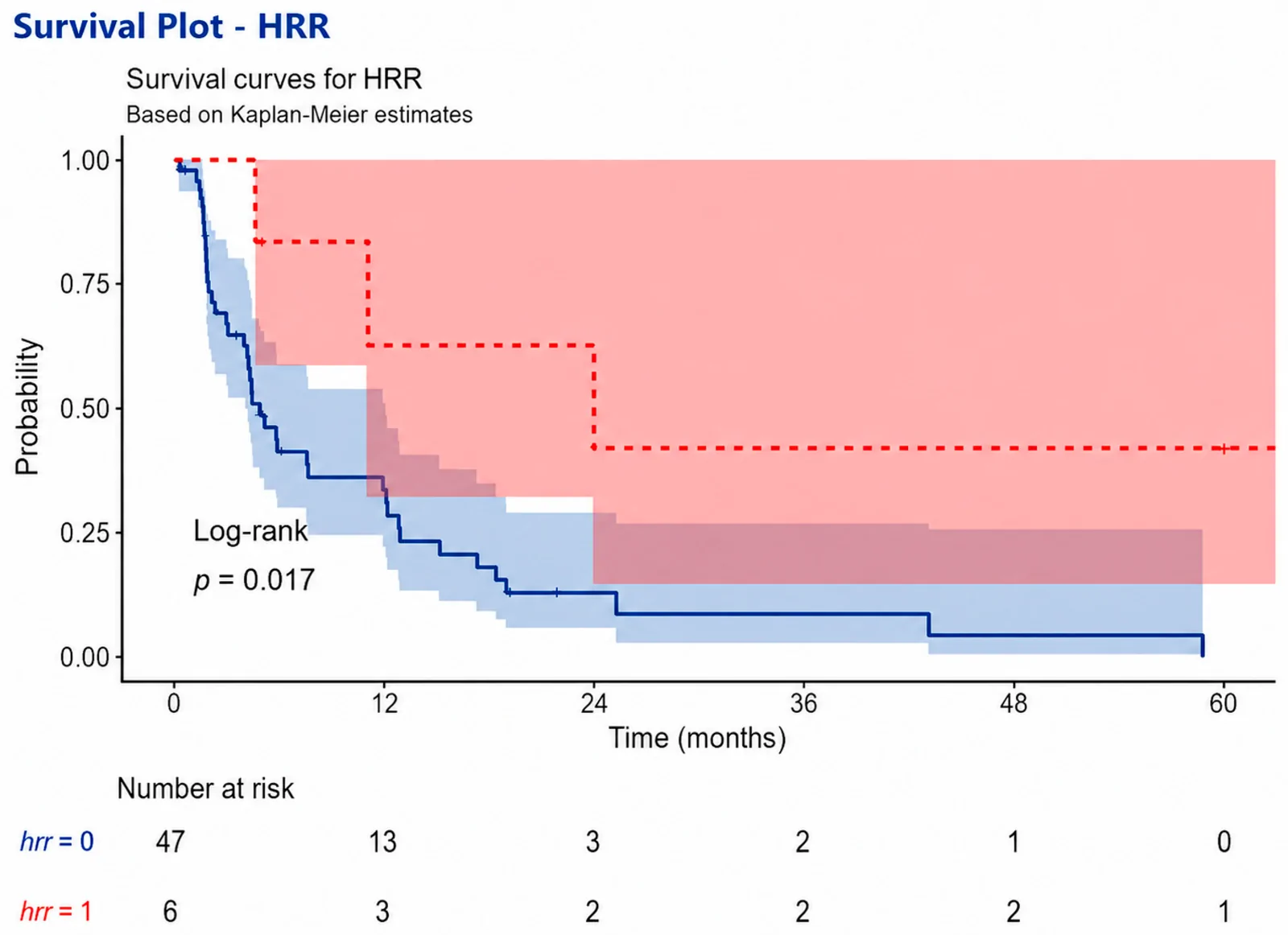
Progression-free survival according to the presence of P/LP germline variants in HRR-associated genes among patients treated with platinum-based chemotherapy.

P/LP germline variants in HRR-associated genes were identified in 20 patients, corresponding to 10.3% of those with available germline results. These variants involved *BRCA2* in nine patients, *ATM* in six, *BRCA1* in two, and *PALB2*, *FANCA*, and *FANCM* in one patient each. Among the 53 patients treated with platinum-based chemotherapy in the metastatic setting, six harbored P/LP germline variants in HRR-associated genes and 47 did not. Median PFS was 23.9 months (*95%* CI, 11.0–NE) in HRR-associated variant carriers versus 4.8 months (*95%* CI, 3.9–12.0) in patients without such variants (log-rank *p* = 0.017), as shown in Figure 4. In univariable Cox regression analysis, HRR-associated P/LP germline variants were associated with a lower risk of progression or death (HR 0.25, *95%* CI, 0.08–0.85; *p* = 0.025). This association remained statistically significant after adjustment for age and platinum administration in the first versus later treatment lines (adjusted HR 0.28, *95%* CI, 0.08–0.96; *p* = 0.042), as shown in Table 1.

Among the 53 patients treated with platinum-based chemotherapy in the metastatic setting, median OS was numerically longer in patients harboring P/LP germline variants in HRR-associated genes than in those without such variants at 33.6 months (*95%* CI, 23.0–NE) versus 18.4 months (*95%* CI, 12.6–39.6), respectively, although the difference was not statistically significant (log-rank *p* = 0.33). Three of six HRR-associated variant carriers and 30 of 47 patients without HRR-associated variants died during follow-up. In univariable Cox regression analysis, HRR-associated variant status was associated with a non-significant reduction in the risk of death (HR 0.56, *95%* CI, 0.17–1.85; *p* = 0.341).

In addition, two patients with germline BRCA1/2 variants subsequently received PARP inhibitor therapy in the metastatic setting, one within a genotype-directed clinical trial in the maintenance setting and one through compassionate use in third-line treatment.

## 4. Discussion

Current NCCN guidelines recommend universal GT for all patients with PDAC, based on the relatively high prevalence of GPVs even among individuals who do not meet traditional referral criteria, as well as the important therapeutic and familial implications of these findings. In contrast, European guidelines have adopted a more conservative approach, generally recommending *BRCA1/2* GT in patients with metastatic disease and broader panel testing individualized according to the clinical context.

Our findings highlight a substantial gap between guideline recommendations and real-world implementation of GT in PDAC, as previously reported in other studies [21,22,23,24]. Despite the growing recognition of the clinical relevance of hereditary predisposition, only 28.9% of patients in our cohort underwent GT. However, a progressive increase in the uptake of GT was observed over time, reaching approximately 65% in both 2024 and 2025. This increase in GT uptake over time was probably multifactorial and should not be attributed exclusively to greater clinician awareness. During the study period, updated European recommendations increased the therapeutic relevance of testing key pancreatic cancer susceptibility genes, institutional referral pathways were revised, testing availability expanded, and the referral pathway changed from a predominantly genetics-led model toward direct test initiation by the oncology team. Under the revised pathway, patients with P/LP germline findings were subsequently referred to Clinical Genetics for post-test counseling and cascade testing. These organizational changes likely reduced the number of steps required before test initiation and facilitated earlier integration of GT into routine oncology care.

The relatively low overall testing rate also reflects disease-specific barriers. PDAC is characterized by an aggressive clinical course and a limited therapeutic window, which may prevent completion of conventional referral pathways involving oncology consultation, genetic counseling, test ordering, and subsequent disclosure of results. Consequently, some patients may never undergo testing or may receive their results only after substantial clinical deterioration or death, limiting both therapeutic utility and opportunities for cascade testing in relatives. Alternative delivery models, including oncologist-led testing, streamlined pre-test counseling, digital educational tools, and testing initiated at diagnosis, may help overcome these barriers. Our experience supports this evolving model of care and suggests that integrating GT directly into the oncology pathway may improve its timely implementation.

Importantly, our study identified a 6.8% rate of P/LP germline variants among patients who did not meet classical referral criteria. These variants involved *ATM* in three patients and *BRCA2*, *CDKN2A*, and *TP53* in one patient each, encompassing genes with potential therapeutic relevance and/or implications for hereditary cancer risk assessment and cascade testing. This observation reinforces one of the key arguments supporting universal testing: the absence of personal or family risk factors does not exclude the presence of P/LP germline variants. Family history and personal-history referral criteria were independently associated with a higher probability of detecting P/LP germline variants. In contrast, younger age was not associated with a higher likelihood of P/LP variant detection. Overall, these clinical characteristics appear insufficient as gatekeepers for germline testing.

The diagnostic yield of P/LP germline variants among tested patients in our cohort was 14.4%, within the range reported in international series. Nevertheless, this estimate should not be interpreted as a population-level prevalence, because GT was performed in a selected subset of the overall cohort and testing strategies evolved over time. Germline variant frequencies may also differ across ethnic and geographic populations, emphasizing the value of generating population-specific real-world data. Despite these considerations, *BRCA2* and *ATM* were the most frequently altered genes in our cohort, followed by *BRCA1*, consistent with the prominent contribution of DNA damage response pathways to hereditary susceptibility in PDAC.

A notable finding was the high proportion of P/LP germline variant carriers with potential therapeutic implications. Twelve of 28 patients (42.9%) harbored P/LP variants in *BRCA1*, *BRCA2*, or *PALB2*, for which there is stronger clinical evidence supporting treatment selection in PDAC. An additional eight patients (28.6%) harbored P/LP variants in *ATM*, *FANCA*, or *FANCM*, which were considered to have potential therapeutic relevance, although the supporting clinical evidence remains limited. Thus, 71.4% of P/LP variant carriers had alterations with either established or potential therapeutic implications. This proportion should not be interpreted as the prevalence of therapeutically relevant alterations in the overall PDAC population, as it refers exclusively to patients in whom a P/LP germline variant was identified. For *BRCA1/2*, the therapeutic relevance is well established, particularly in relation to platinum sensitivity and PARP inhibition; in the POLO trial, maintenance olaparib prolonged PFS from 3.8 to 7.4 months in platinum-sensitive metastatic PDAC with germline BRCA1/2 variants, although no significant OS benefit was demonstrated [4,25,26]. Clinical evidence also supports platinum- and PARPi-based strategies in *PALB2*-mutated PDAC [4,26].

In contrast, the predictive significance of non-core HRR alterations remains less certain. Pishvaian et al. included *ATM* and Fanconi pathway genes within a broader HR-DDR group and observed improved outcomes with platinum exposure in advanced HR-DDR-mutated PDAC, although subgroup sizes were insufficient to establish the predictive value of individual non-*BRCA* genes [31]. Similarly, Park et al. found that core *BRCA1/2/PALB2* alterations and, particularly, biallelic HRR inactivation were associated with greater genomic instability and appeared to enrich for platinum sensitivity, suggesting that germline variant status alone may not establish functional tumor HRD [33]. This distinction may be particularly relevant for *ATM*, which may not consistently confer a canonical HRD phenotype or platinum sensitivity [34]. Evidence for *FANC* genes also remains limited despite their biological involvement in DNA repair; notably, tumor genomic profiling in the patient harboring a germline *FANCM* variant independently reported an HRD-positive phenotype. These findings support integrating germline results with tumor-level evidence of biallelic inactivation, loss of heterozygosity, or genomic HRD signatures whenever available [9].

The distinction between molecular therapeutic relevance and actual therapeutic impact was also clinically relevant. Among the 20 patients with variants of established or potential therapeutic relevance, 11 (55.0%) received matched therapy, whereas knowledge of the germline result specifically led to treatment modification in only six patients (30.0%). Some patients had already received a biologically concordant platinum-containing regimen before the germline result became available, whereas others were unable to receive matched treatment because of frailty, platinum contraindication, rapid clinical deterioration, or because the result became available after death. Two patients with germline *BRCA1/2* variants also received PARP inhibitor therapy, one as maintenance following platinum-based chemotherapy and the other after progression on previous treatment lines. Access to PARP inhibition was additionally limited by the lack of reimbursement of olaparib for this indication within the Spanish National Health System during the study period. These observations illustrate that identifying a therapeutically relevant germline alteration does not necessarily translate into treatment modification and reinforce the importance of obtaining GT results early in the disease course.

Our exploratory survival findings are consistent with the hypothesis that P/LP germline variants in HRR-associated genes may identify a subgroup with favorable outcomes when treated with DNA-damaging therapies. Among platinum-treated patients, carriers of P/LP variants in HRR-associated genes had significantly longer PFS than patients without such variants, and median OS was also numerically longer, although the difference was not statistically significant. Importantly, outcomes among HRR-associated variant carriers were heterogeneous: while some patients experienced prolonged disease control and survival, others progressed considerably earlier. This variability suggests that the potential benefit associated with HRR-directed treatment is not uniform and that a subset of patients may derive particularly durable benefit. Identifying the molecular and clinical characteristics that distinguish these long-term responders warrants further investigation. These observations are directionally consistent with previous studies. Pishvaian et al. reported a median OS of 2.37 years in advanced HR-DDR-mutated PDAC treated with platinum compared with 1.45 years in HR-DDR-proficient tumors, whereas no corresponding survival advantage was observed in platinum-naïve patients [31].

However, our findings should not be interpreted as evidence of a platinum-specific predictive effect. One *BRCA1/2* carrier received PARPi maintenance following platinum-based chemotherapy and another received PARP inhibition at a later stage after progression on previous lines. In the former patient, PARP inhibition may have contributed to both PFS and OS, whereas later-line PARPi exposure in the latter may have influenced OS. Therefore, the favorable outcomes observed in this small subgroup may reflect the effect of a broader sequentially matched therapeutic strategy rather than platinum chemotherapy alone. Accordingly, these survival analyses should be considered exploratory and hypothesis-generating.

This study has several limitations. Its retrospective design entails inherent risks of selection bias, missing data, and incomplete ascertainment of personal and family cancer history. GT strategies were heterogeneous in terms of gene content, sequencing platform, CNV assessment, and bioinformatic workflow, reflecting the evolution of real-world testing during the study period but limiting direct comparability across patients. Although the most frequently used 12-gene panel included the main PDAC susceptibility genes, broader panels enabled the detection of P/LP variants in additional genes not included in the core panel; therefore, the frequency of P/LP variants in less frequently tested genes may have been underestimated. In addition, GT was performed in a selected subset of the overall cohort, and the reasons for testing or non-testing, including test availability and clinician-related factors, were not systematically collected. Therefore, the diagnostic yield among tested patients should not be interpreted as a population-level prevalence estimate, and the relative contribution of different factors to the temporal increase in testing could not be quantified.

The exploratory survival analyses were limited by the small number of patients with P/LP variants in HRR-associated genes who received platinum-based chemotherapy at the survival-analysis data cutoff (n = 6), treatment heterogeneity, and limited ability to perform robust multivariable or regimen-specific analyses. Treatment allocation was not randomized and may have been influenced by performance status, comorbidities, and disease trajectory, introducing potential confounding by indication. Relevant prognostic variables, including baseline ECOG performance status and CA19-9 levels, were not systematically available for inclusion in the multivariable models, and residual confounding cannot therefore be excluded. Detailed measures of treatment exposure and toxicity were not systematically collected. Moreover, two BRCA1/2 carriers received PARP inhibitors during their disease course, one as maintenance after platinum and one at a later treatment line; therefore, the contribution of platinum chemotherapy and PARP inhibition to survival outcomes could not be assessed separately. Tumor-level assessment of HRD was not systematically available, except in one *FANCM* carrier, limiting our ability to determine the functional significance of individual germline HRR-associated variants. Accordingly, the survival associations should be regarded as exploratory and hypothesis-generating and require validation in larger prospective cohorts.

Despite these limitations, this study provides real-world data from a large cohort of patients with PDAC, including a substantial proportion who did not meet classical referral criteria. It also reflects the progressive implementation of GT over a prolonged period of evolving clinical recommendations and testing pathways. The identification of P/LP variants in patients without traditional referral criteria, together with the substantial proportion of variant carriers with established or potential therapeutic implications, supports earlier and broader integration of GT into routine PDAC care. The survival findings should be considered exploratory but provide additional rationale for prospective evaluation of treatment strategies according to HRR-associated germline variant status.

## 5. Conclusions

In summary, P/LP germline variants were identified in a clinically relevant proportion of tested patients with PDAC in this Spanish cohort, including individuals who did not meet classical referral criteria. Earlier and more systematic implementation of GT may improve the identification of variants with therapeutic and familial implications and facilitate timely treatment decisions, genetic counseling, and cascade testing. The exploratory survival findings suggest that P/LP germline variants in HRR-associated genes may be associated with favorable outcomes with platinum-based therapy, but these observations require validation in larger prospective cohorts.

## Figures and Tables

**Table 1:** Univariable and multivariable Cox regression analyses of factors associated with progression-free survival among patients receiving platinum-based chemotherapy in the metastatic setting.

Variable	Univariable HR (*95% CI*)	*p*-Value	Adjusted HR (*95% CI*)	*p*-Value
HRR-associated P/LP germline variant (yes vs. no)	0.25 (0.08–0.85)	0.025	0.28 (0.08–0.96)	0.042
Platinum treatment line (later vs. first line)	2.77 (1.44–5.33)	0.002	2.81 (1.38–5.72)	0.004
Age (per 1-year increase)	1.00 (0.97–1.04)	0.946	0.97 (0.94–1.01)	0.160

HR, hazard ratio; CI, confidence interval; HRR, homologous recombination repair; P/LP, pathogenic/likely pathogenic. The multivariable model included HRR-associated germline variant status, age, and platinum administration in the first versus later treatment lines.

## Data Availability

The data supporting the findings of this study are available from the corresponding author upon reasonable request. The data are not publicly available due to privacy and ethical restrictions related to patient-level clinical and genetic information.

## Supplementary Materials

The following supporting information can be downloaded at: https://www.mdpi.com/article/10.3390/cancers18162580/s1, Supplementary Table S1: Platinum-containing regimens; Supplementary Table S2: Exact gene composition of the germline testing strategies; Supplementary Table S3: Technical characteristics of the multigene germline testing strategies; Supplementary Table S4: Baseline characteristics according to germline testing status; Supplementary Table S5: Therapeutic implications of pathogenic/likely pathogenic germline variants; Supplementary Table S6: Distribution of platinum-containing regimens according to treatment line.
