# Supplementary material for: Real-World Germline Testing Patterns and Clinical Implications of HRR-Associated Germline Variants in Pancreatic Ductal Adenocarcinoma
Source: Cancers (Basel). 2026 Aug 11;18(16):2580. doi: 10.3390/cancers18162580 (PMC13511406; doi:10.3390/cancers18162580)
Supplement: Supplementary file 1 [file cancers-18-02580-s001.zip › cancers-4437006-supplementary.pdf]

Supplementary Tables

Supplementary Table S1. Platinum-containing regimens.

Regimen	Drugs and planned doses	Schedule
FOLFIRINOX	Oxaliplatin 85 mg/m^2; irinotecan 180 mg/m^2; leucovorin 400 mg/m^2; 5-FU bolus 400 mg/m^2 + 2400 mg/m^2/46 h	Every 14 days
Modified FOLFIRINOX	Oxaliplatin 85 mg/m^2; irinotecan 150 mg/m^2; leucovorin 400 mg/m^2; 5-FU 2400 mg/m^2/46 h	Every 14 days
NALIRIFOX	Liposomal irinotecan 50 mg/m^2; oxaliplatin 60 mg/m^2; leucovorin 400 mg/m^2; 5-FU 2400 mg/m^2/46 h	Days 1 and 15 of each 28-day cycle
Cisplatin + gemcitabine	Cisplatin 25 mg/m^2; gemcitabine 1000 mg/m^2	Days 1 and 8 of each 21-day cycle
SEQUENCE	Nab-paclitaxel 125 mg/m^2 + gemcitabine 1000 mg/m^2, followed by mFOLFOX6	Nab-paclitaxel + gemcitabine on days 1, 8, and 15, followed by mFOLFOX6 on day 29 of each 42-day cycle
mFOLFOX6	Oxaliplatin 85 mg/m^2; leucovorin 400 mg/m^2; 5-FU bolus 400 mg/m^2 + 2400 mg/m^2/46 h	Every 14 days

Supplementary Table S2. Exact gene composition of the germline testing strategies.

These tables summarize the gene composition and technical characteristics of the germline testing strategies used during the study period. The testing strategy was selected and, when clinically indicated, expanded according to the patient's clinical presentation, personal and family history, and suspected hereditary cancer syndrome.

Testing strategy	Period	Genes, n	Exact gene composition and additional analyses
**Targeted familial variant testing**	According to clinical indication	**1**	Analysis was restricted to the gene harboring a previously identified *BRCA2* P/LP germline variant in a first-degree relative.
**Hereditary pancreatic cancer panel (HUMS, pre-2025)**	Before 2025	**12**	**Genes:** *APC, ATM, BRCA1, BRCA2, CDKN2A, MLH1, MRE11A, MSH2, PALB2, PMS2, STK11, TP53*<br>**Additional analysis:** Bioinformatic CNV analysis; additional MLPA deletion/duplication analysis in BRCA1, BRCA2, and ATM.
**Hereditary pancreatic cancer panel (HUMS, 2025 onward)**	From 2025	**13**	**Genes:** *APC, ATM, BRCA1, BRCA2, CDKN2A, EPCAM, MLH1, MSH2, MSH6, PALB2, PMS2, STK11, TP53*<br>**Additional analysis:** Bioinformatic CNV analysis; suspected or clinically relevant CNVs were confirmed by MLPA and/or CGH array.
**Breast-ovarian cancer panel (HUMS, pre-2024)**	Before 2024	**21**	**Genes:** *APC, ATM, BRCA1, BRCA2, BRIP1, CDH1, CHEK2, ERCC4, HOXB13, MLH1, MSH2, MSH6, MUTYH, PALB2, PMS1, PTEN, RAD51C, RAD51D, STK11, TP53, XRCC2*<br>**Additional analysis:** The same pre-2024 laboratory workflow as the pancreatic cancer panel was used.
**Breast-ovarian cancer panel (HUMS, post-2024)**	2024/2025 onward.	**26**	**2024/2025 composition (26 genes):** *APC, ATM, BARD1, BRCA1, BRCA2, BRIP1, CDH1, CHEK2, ERCC4, HOXB13, MLH1, MRE11, MSH2, MSH6, MUTYH, NBN, PALB2, PMS1, PMS2, PTEN, RAD50, RAD51C, RAD51D, STK11, TP53, XRCC2*<br>**Additional analysis:** Bioinformatic CNV analysis; suspected or clinically relevant CNVs were confirmed by MLPA and/or CGH array.
**Hereditary cancer panel (Sistemas Genómicos)**	Before 2024	**111**	**Genes:** *ACD, AIP, APC, ATM, ATR, AXIN2, BAP1, BARD1, BLM, BMPR1A, BRCA1, BRCA2, BRIP1, BUB1, CDH1, CDK4, CDKN1B, CDKN2A, CHEK2, DDB2, DICER1, DIS3L2, DKC1, ELANE, EPAS1, EPCAM, ERCC1, ERCC2, ERCC3, ERCC4, ERCC5, ERCC6, FANCA, FANCB, FANCC, FANCD2, FANCE, FANCF, FANCG, FANCI, FANCL, FANCM, FH, FLCN, G6PC3, GFI1, GREM1, HOXB13, JAGN1, KIF1B, KIT, MAX, MDH2, MEN1, MET, MITF, MLH1, MNX1, MSH2, MSH6, MSR1, MUTYH, NBN, NF2, NFIX, NHP2, NOP10, NSD1, NTHL1, PALB2, PARN, PDGFRA, PMS1, POLD1, POLE, POLH, POT1, PRKAR1A, PTCH1, PTEN, RAD50, RAD51, RAD51C, RAD51D, RB1, RECQL, RECQL4, RET, RTEL1, SCG5, SLX4, SMAD4, SMARCA4, STK11, SUFU, TERC, TERT, TINF2, TMEM127, TP53, TSC1, TSC2, UBE2T, VHL, VPS45, WAS, WRN, WT1, XPA, XPC, XRCC2*<br>**Additional analysis:** P/LP variants were confirmed by Sanger sequencing; VUS were not routinely confirmed.
**Hereditary cancer panel (HUMS)**	2024/2025 onward	**115**	**Genes:** *AIP, APC, ATM, ATR, AXIN2, BAP1, BARD1, BLM, BMPR1A, BRCA1, BRCA2, BRIP1, BUB1, CDH1, CDK4, CDKN1B, CDKN2A, CHEK2, CTNNA1, DDB2, DICER1, DIS3L2, ELAC2, EPAS1, EPCAM, ERCC1, ERCC2, ERCC3, ERCC4, ERCC5, ERCC6, ABRAXAS1, FANCA, FANCB, FANCC, FANCD2, FANCE, FANCF, FANCG, FANCI, FANCL, FANCM, FH, FLCN, GEN1, GREM1, HNF1A, HNF1B, HOXB13, KIF1B, KIT, MAX, MC1R, MDH2, MEN1, MET, MITF, MLH1, MNX1, MRE11, MSH2, MSH6, MSR1, MUTYH, NBN, NF1, NF2, NFIX, NSD1, NTHL1, PALB2, PDGFRA, PMS1, PMS2, POLD1, POLE, POLH, POT1, PRKAR1A, PRSS1, PTCH1, PTEN, RAD50, RAD51, RAD51C, RAD51D, RAD54L, RB1, RECQL, RECQL4, RET, RNASEL, SCG5, SDHA, SDHAF2, SDHB, SDHC, SDHD, SLX4, SMAD4, SMARCA4, STK11, SUFU, TMEM127, TP53, TSC1, TSC2, UBE2T, VHL, WRN, WT1, XPA, XPC, XRCC2, XRCC3*<br>**Additional analysis:** Bioinformatic CNV analysis; confirmation by MLPA and/or CGH array when indicated.

CGH, comparative genomic hybridization; **CNV**, copy number variation; **HUMS**, Miguel Servet University Hospital; **MLPA**, multiplex ligation-dependent probe amplification; **P/LP**, pathogenic/likely pathogenic; **VUS**, variant of uncertain significance.

Supplementary Table S3. Technical characteristics of the multigene germline testing strategies.

Testing strategy	Laboratory and period	Library preparation and sequencing platform	Reference genome and analysed regions	Analytical thresholds and coverage	CNV analysis, confirmation, interpretation, and main limitations
**Hereditary pancreatic cancer panel (HUMS, pre-2025)**	Clinical Genetics Section, Miguel Servet University Hospital; before 2025	Illumina NGS platform according to the year of testing; panel-based or exome-based capture workflows were used during the study period.	Coding exons and adjacent intronic regions; transcript-specific reference sequences	Read depth ≥20× and variant allele fraction ≥0.20	Bioinformatic CNV analysis and Sanger confirmation of clinically relevant variants were performed. Large deletions and duplications in BRCA1, BRCA2, and ATM were assessed by MLPA using MRC-Holland probes and ABI3130XL fragment analysis. Main limitations included high-GC or repetitive regions, insertions >4 bp, deletions >11 bp, homologous regions, deep-intronic variants, and analytical sensitivity below 100%.
**Hereditary pancreatic cancer panel (HUMS, post-2025)**	Clinical Genetics Section, Miguel Servet University Hospital; from 2025	Automated NGS Clinical STARlet library preparation; Illumina DNA Prep with Enrichment Dx and Illumina Exome 2.5 + Twist Bioscience for Illumina Mitochondrial Panel; Illumina NovaSeq 6000 Dx; paired-end sequencing	GRCh38; coding exons and adjacent intronic regions up to ±10 bp	Read depth ≥10× and variant allele fraction ≥0.20.	Bioinformatic CNV analysis; clinically relevant low-quality variants were confirmed by Sanger sequencing. CNVs were confirmed by MLPA and/or CGH array when indicated. Variant interpretation was performed using VarSome Clinical (CE-IVD) and complementary in silico tools. Main limitations included low-coverage or complex regions, insertions >10–12 nt, deletions >25 nt, homologous and deep-intronic regions, excluded homopolymers ≥8 bp, low-level mosaicism, and analytical sensitivity below 100%
**Breast-ovarian cancer panel (HUMS, pre-2024)**	Clinical Genetics Section, Miguel Servet University Hospital; before 2024	Illumina-based NGS using the library preparation, capture workflow, and sequencing platform available at the time of testing; paired-end sequencing. The general laboratory workflow was the same as that used for the contemporaneous pancreatic cancer panel, with a different gene composition.	Coding exons and adjacent intronic regions; transcript-specific reference sequences	Read depth ≥20× and variant allele fraction ≥0.20	Bioinformatic CNV analysis and Sanger confirmation of clinically relevant variants were performed according to the contemporaneous laboratory protocol. Specific orthogonal CNV confirmation procedures were not separately documented for this panel. Main limitations included high-GC or repetitive regions, homologous regions, larger insertions or deletions, deep-intronic variants, and analytical sensitivity below 100%
**Breast-ovarian cancer panel (HUMS, post-2024)**	Clinical Genetics Section, Miguel Servet University Hospital; 2024/2025 onward.	Automated NGS Clinical STARlet library preparation; Illumina DNA Prep with Enrichment Dx and Illumina Exome 2.5 + Twist Bioscience for Illumina Mitochondrial Panel; Illumina NovaSeq 6000 Dx; paired-end sequencing	GRCh38; coding exons and adjacent intronic regions up to ±10 bp	Read depth ≥10× and variant allele fraction ≥0.20.	Bioinformatic CNV analysis; clinically relevant low-quality variants were confirmed by Sanger sequencing. CNVs were confirmed by MLPA and/or CGH array when indicated. Variant interpretation was performed using VarSome Clinical (CE-IVD) and complementary in silico tools. Main limitations included low-coverage or complex regions, larger insertions or deletions, homologous and deep-intronic regions, excluded homopolymers, low-level mosaicism, and analytical sensitivity below 100%.
**Hereditary cancer panel (Sistemas Genómicos)**	Sistemas Genómicos, Spain; before 2024	Solution-based hybrid capture; Illumina HiSeq; paired-end sequencing	GRCh38; coding regions and intronic flanking regions up to ±8 bp	Read depth ≥20× and variant allele fraction ≥0.20	P/LP variants were confirmed by Sanger sequencing; VUS were not routinely confirmed. CNV analysis was not explicitly documented for this strategy. Main limitations included complex or homologous regions, insertions >10–12 bp, deletions >25 bp, deep-intronic variants, large rearrangements, and low-level mosaicism.
**Hereditary cancer panel (HUMS)**	Clinical Genetics Section, Miguel Servet University Hospital; 2024/2025 onward	Automated NGS Clinical STARlet library preparation; Illumina DNA Prep with Enrichment Dx and Illumina Exome 2.5 + Twist Bioscience for Illumina Mitochondrial Panel; Illumina NovaSeq 6000 Dx; paired-end sequencing	GRCh38; coding exons and adjacent intronic regions up to ±10 bp	Read depth ≥10× and variant allele fraction ≥0.20	Bioinformatic CNV analysis; clinically relevant low-quality variants were confirmed by Sanger sequencing. CNVs were confirmed by MLPA and/or CGH array when indicated. Variant interpretation was performed using VarSome Clinical (CE-IVD) and complementary in silico tools. Main limitations included low-coverage or complex regions, insertions >10–12 nt, deletions >25 nt, homologous and deep-intronic regions, excluded homopolymers ≥8 bp, low-level mosaicism, and analytical sensitivity below 100%.

Abbreviations: bp, base pairs; **CE-IVD**, Conformité Européenne–in vitro diagnostic; **CGH**, comparative genomic hybridization; **CNV**, copy number variation; **GRCh38**, Genome Reference Consortium Human Build 38; **HUMS**, Miguel Servet University Hospital; **MLPA**, multiplex ligation-dependent probe amplification; **NGS**, next-generation sequencing; **nt**, nucleotides; **P/LP**, pathogenic/likely pathogenic; **VUS**, variant of uncertain significance.

General laboratory platform evolution at Miguel Servet University Hospital: Illumina MiSeq was used from 2016 to 2019 for panel-based workflows (Onco-GeneSGKit X and Oncoplus; Sistemas Genómicos); Illumina NextSeq was used from 2019 to 2023 for complete-exome workflows (Exome-GeneSGKit X Auto; Sistemas Genómicos); and Illumina NovaSeq Dx has been used from 2024 onward for complete-exome sequencing with Illumina Exome 2.5 + Twist Bioscience for Illumina Mitochondrial Panel. Despite platform changes, mean gene coverage remained broadly homogeneous at approximately 300×. Panel size varied over time, and genes included only in broader panels were consequently not assessed uniformly across the entire cohort.

Supplementary Table S4. Baseline characteristics according to germline testing status

Characteristic	No GT (n=479)	GT (n=195)	p-value
**Age at diagnosis, mean (SD), years**	67.5 (9.5)	64.7 (10.8)	**0.001**
**Age ≤60 years, n (%)**	109 (22.8%)	69 (35.4%)	**<0.001**
**Sex, n (%)**			**0.500**
Female	227 (47.4%)	98 (50.3%)	
Male	252 (52.6%)	97 (49.7%)	
**Stage at diagnosis, n (%)**			**0.200**
Resectable	109 (22.8%)	38 (19.5%)	
Borderline resectable	27 (5.6%)	16 (8.2%)	
Locally advanced	67 (14.0%)	19 (9.7%)	
Metastatic	276 (57.6%)	122 (62.6%)	
**Family history referral criterion, n (%)**	3 (0.6%)*	36 (18.5%)	**<0.001**
**Personal history referral criterion, n (%)**	5 (1.0%)*	17 (8.7%)	**<0.001**

Abbreviations: GT, germline testing; SD, standard deviation.
* Percentages were calculated using available data; family and personal history information was missing for one patient in the non-GT group.
Statistical analysis: Continuous variables were compared using Welch's t-test when the assumption of equal variances was violated. Categorical variables were compared using the chi-square test.

Supplementary Table S5. Therapeutic implications of pathogenic/likely pathogenic germline variants

Pt	Gene	Established/potential therapeutic relevance	Matched therapy	Treatment changed based on germline result	Platinum-based chemotherapy	Disease setting at platinum treatment	PARP inhibitor	Genotype-directed clinical trial	Reason for no matched therapy
1	**BRCA2**	Yes	**No**	No	No		**No**	No	Clinical frailty when the germline result became available
2	**PALB2**	Yes	**Yes**	No	Yes	Metastatic		No	
3	**FANCA**	Yes	**No**	No	No			No	Germline result became available after death
4	**ATM**	Yes	**No**	No	No			No	Clinical frailty when the germline result became available
5	**ATM**	Yes	**Yes**	**Yes**	Yes	Both (adjuvant and metastatic)		No	
6	**BRCA2**	Yes	**Yes**	**Yes**	Yes	Metastatic	**No**	No	
7	**BRCA2**	Yes	**Yes**	No	Yes	Adjuvant		No	
8	**ATM**	Yes	**No**	No	No			No	Ongoing clinical benefit from another treatment; treatment modification was deferred.
9	**STK11**	**No**							
10	**ATM**	Yes	**Yes**	**No**	Yes	Adjuvant		No	
11	**ATM**	Yes	**No**	No	No			No	Germline result became available after death
12	**BRCA2**	Yes	**No**	No	No		**No**	**No**	Ongoing clinical benefit from a non-genotype-directed clinical trial when the germline result became available; subsequent clinical deterioration after progression precluded platinum therapy
13	**BRCA2**	Yes	**Yes**	**Yes**	Yes	Metastatic	**No**	No	
14	**BRCA2**	Yes	**Yes**	**Yes**	Yes	Metastatic	**Yes**	**Yes**	PARP inhibitor administered as maintenance therapy following platinum-based chemotherapy within a genotype-directed clinical trial.
15	**TP53**	**No**							
16	**FANCM**	**Yes†**	**Yes**	**No**	Yes	Adjuvant		No	
17	**MUTYH**	**No**							
18	**BRCA2**	Yes	**Yes**	No	Yes	Adjuvant		No	
19	**BRCA2**	Yes	**No**	No	No		**No**	No	Clinical frailty when the germline result became available
20	**BRCA1**	Yes	**Yes**	**Yes**	Yes	Adjuvant		No	
21	**ATM**	Yes	**No**	No	No			No	Platinum-based chemotherapy was contraindicated because of clinical frailty
22	**RECQL4**	**No**							
23	**MUTYH**	**No**							

Pt	Gene	Established/potential therapeutic relevance	Matched therapy	Treatment changed based on germline result	Platinum-based chemotherapy	Disease setting at platinum treatment	PARP inhibitor	Genotype-directed clinical trial	Reason for no matched therapy
24	**CDKN2A**	**No**							
25	**STK11**	**No**							
26	**BRCA2**	Yes	**No**	No	No		**No**	No	Germline result became available at the time of death
27	**BRCA1**	Yes	**Yes**	**Yes**	Yes	Metastatic	**Yes‡**	**No**	
28	**HOXB13**	**No**							

† In patient 16, comprehensive tumor genomic profiling additionally confirmed an HRD-positive status.

‡ PARP inhibitor administered in the metastatic setting through compassionate use. No genotype-directed clinical trial was available in Spain at that time.

Variants of established or potential therapeutic relevance were defined as P/LP germline variants with established or potential therapeutic implications in pancreatic cancer. For this descriptive analysis, matched therapy was defined as receipt, at any time during the disease course, of a treatment considered biologically concordant with the identified germline variant, including platinum-based chemotherapy, PARP inhibition, or genotype-directed clinical trial treatment. Platinum administered in the adjuvant setting was therefore considered matched therapy. Treatment modification based on the germline result was assessed separately and required that knowledge of the germline finding directly influenced the therapeutic decision. Treatment-related fields were left blank for patients without variants of established or potential therapeutic relevance.

Supplementary Table S6. Distribution of platinum-containing regimens according to treatment line.

Platinum-containing regimen	First line	Second line	Third line	Total, n (%)
FOLFIRINOX/mFOLFIRINOX	21	1	1	23 (43.4%)
NALIRIFOX	6	8	1	15 (28.3%)
Cisplatin–gemcitabine	2	0	2	4 (7.5%)
SEQUENCE	4	0	0	4 (7.5%)
FOLFOX6	0	2	5	7 (13.2%)
**Total**	**33 (62.3%)**	**11 (20.8%)**	**9 (17.0%)**	**53 (100%)**
